# An mHealth App to Promote Adherence to Immunosuppressant Medication and Track Symptoms in Children After Hematopoietic Stem Cell Transplant: Protocol for a Mixed Methods Usability Study

**DOI:** 10.2196/39098

**Published:** 2022-07-21

**Authors:** Micah Skeens, Emre Sezgin, Jack Stevens, Wendy Landier, Ahna Pai, Cynthia Gerhardt

**Affiliations:** 1 Center for Biobehavioral Health Abigail Wexner Research Institute Nationwide Children's Hospital Columbus, OH United States; 2 Nationwide Children's Hospital Columbus, OH United States; 3 Division of Pediatric Hematology Oncology University of Alabama at Birmingham Birmingham, AL United States; 4 Department of Pediatrics University of Cincinnati College of Medicine Cincinnati, OH United States

**Keywords:** medication adherence, digital health, mHealth, pediatrics, app, bone marrow transplant, adherence, usability, feasibility, caregivers, children, hematopoietic stem cell transplant, HSCT

## Abstract

**Background:**

In the United States, poor adherence accounts for up to 70% of all medication-related hospital admissions, resulting in $100 billion in health care costs annually. In pediatrics, adherence is largely dependent on caregivers. In a high-risk hematopoietic stem cell transplant (HSCT) population, caregivers are isolated with their child due to infection risk and must manage challenging treatment regimens at home, often with limited time and support. Complex behavioral interventions, typically employed to address adherence, are difficult to deliver and manage in the context of these daily tasks. The most successful adherence interventions, and thus improved clinical outcomes, have included mobile health (mHealth) reminder approaches and a direct measure of adherence.

**Objective:**

This is a 3-phase project, with this protocol describing phase 2, to determine the usability and feasibility of an mHealth app (BMT4me) designed to promote adherence to immunosuppressant medication and to track symptoms among children who received HSCT.

**Methods:**

This study uses an iterative convergent mixed methods design to develop and assess the usability and feasibility of an adherence digital health intervention. We will recruit 15 caregivers of pediatric patients receiving HSCT to complete user testing. Qualitative and quantitative data will be integrated to enhance and expand upon study findings.

**Results:**

Enrollment began in September 2021 and is ongoing. A total of 7 caregivers have enrolled. We anticipate completion by fall 2022. We anticipate high usability scores and a better understanding of unique features within the app that are needed for HSCT families post transplant. To date, usability scores among enrolled participants are greater than 70%. Feedback from qualitative interviews is being used to further adapt the app by adding specific weekly logs, call provider options, and voice to text.

**Conclusions:**

This protocol describes a mixed methods usability and feasibility study to develop and implement a smartphone app for caregivers of children receiving HSCT. The app was designed to improve immunosuppressant adherence and to track symptoms in the acute phase post discharge. Study findings will inform further refinement of the app and the feasibility of a pilot randomized controlled trial examining efficacy on clinical outcomes.

**Trial Registration:**

ClinicalTrials.gov NCT04976933; https://clinicaltrials.gov/ct2/show/NCT04976933

**International Registered Report Identifier (IRRID):**

DERR1-10.2196/39098

## Introduction

### Background

In the United States, poor adherence accounts for up to 70% of all medication-related hospital admissions [1], resulting in $100 billion in health care costs annually [2]. An estimated 50% to 80% of pediatric patients are nonadherent to medications [3-6]. Reasons for nonadherence are multifactorial. The most important determinants of nonadherence are consistently documented as complexity and duration of treatment regimens, as well as forgetfulness [7-9]. Thus, children undergoing difficult hematopoietic stem cell transplants (HSCTs) who require medication indefinitely are at high risk for medication nonadherence.

HSCT is the transplantation of hematopoietic stem cells derived from bone marrow (ie, “bone marrow transplant” or peripheral blood stem cells). HSCT became the common treatment for life-threatening malignant (eg, leukemia, lymphoma) and nonmalignant (eg, aplastic anemia, sickle cell disease, immune deficiencies) disorders in the mid-1900s. Recipients of bone marrow transplants must adhere to multifaceted outpatient regimens that require strict personal hygiene, environmental restrictions, and complex medication regimens. These include wearing masks, avoiding large crowds, frequent mouth care, and taking multiple time-sensitive medications throughout the day.

Lack of adherence to any of these regimens can be life-threatening [10]. Adherence to immunosuppressant medications during the acute phase (ie, the first 100 days) post transplant is critical to prevent graft versus host disease and avoid graft failure. Studies in children with HSCT report suboptimal adherence rates (52%-78%) that worsen over time [11-13]. Nonadherence to oral medications has been associated with a greater incidence of infections in children during the outpatient HSCT treatment phase [12]. Providers also report adherence as a major concern for outpatient pediatric HSCT recipients [14]. However, no studies have examined adherence specifically to immunosuppressant medications, which are key to engraftment and ultimately survival.

Multiple factors influence adherence, but, ultimately, the final common pathway to adherence is human behavior [15]. For children, caregivers are ultimately responsible for adherence, including refilling prescriptions, retrieving medications, and administering them correctly. In a high-risk HSCT population, caregivers are susceptible to fatigue and stress from being isolated with their child due to infection risk and independently managing complex treatment regimens at home, often with limited time and support. Unfortunately, behavioral interventions employed to address adherence are often difficult to deliver and manage in the context of these daily tasks. Specifically, these interventions can involve 8 to 12 face-to-face sessions, tracking behaviors, practicing skills, and other homework, which can be overwhelming to caregivers.

Alternatively, behavioral economics (BE) theory suggests that small “nudges” can produce and sustain behavior change [16]. A BE approach is a significant paradigm shift from the complex cognitive behavioral theories that inform most adherence interventions. Instead, BE assumes decision-making can be influenced through low-intensity interventions to lead patients to optimal choices [17]. Adherence to medication and exercise programs using BE-designed interventions in adults have some initial promising data [18-21]. Within pediatrics, for example, BE has been successful in increasing fruit consumption and improving vaccination rates [22-24].

With mobile health (mHealth) access nearly ubiquitous and an estimated 250 billion mHealth app downloads in 2022 [25], technology has great potential to improve adherence [26]. mHealth interventions based on BE principles may be ideal, as they provide sophisticated yet simple “nudges” to individuals [27]. Such reminder apps target forgetfulness, a common reason for poor adherence [28,29]. These mHealth interventions are ideally suited for complex HSCT regimens requiring multiple-dose medications that are time sensitive.

A recent systematic review in solid organ transplant reported the most successful interventions, resulting in increased adherence and improved clinical outcomes (eg, complications, hospital admissions, survival) have used mHealth reminders and a direct measure of adherence [30]. Although direct measures of adherence (eg, serum assays) can avoid bias inherent in self- or parent reports, no studies have used direct measures of adherence or mHealth interventions to promote immunosuppression adherence in the acute phase after an HSCT (ie, discharge to day 100). Furthermore, the feasibility, acceptability, and efficacy of BE-designed mHealth interventions have not been examined in the HSCT population. Thus, this study utilizes a BE approach to mHealth app development, as well as an iterative approach of cyclical evaluation and revision, to maximize the usability and adoption of the digital intervention, BMT4me.

### BMT4me

**Figure 1:**
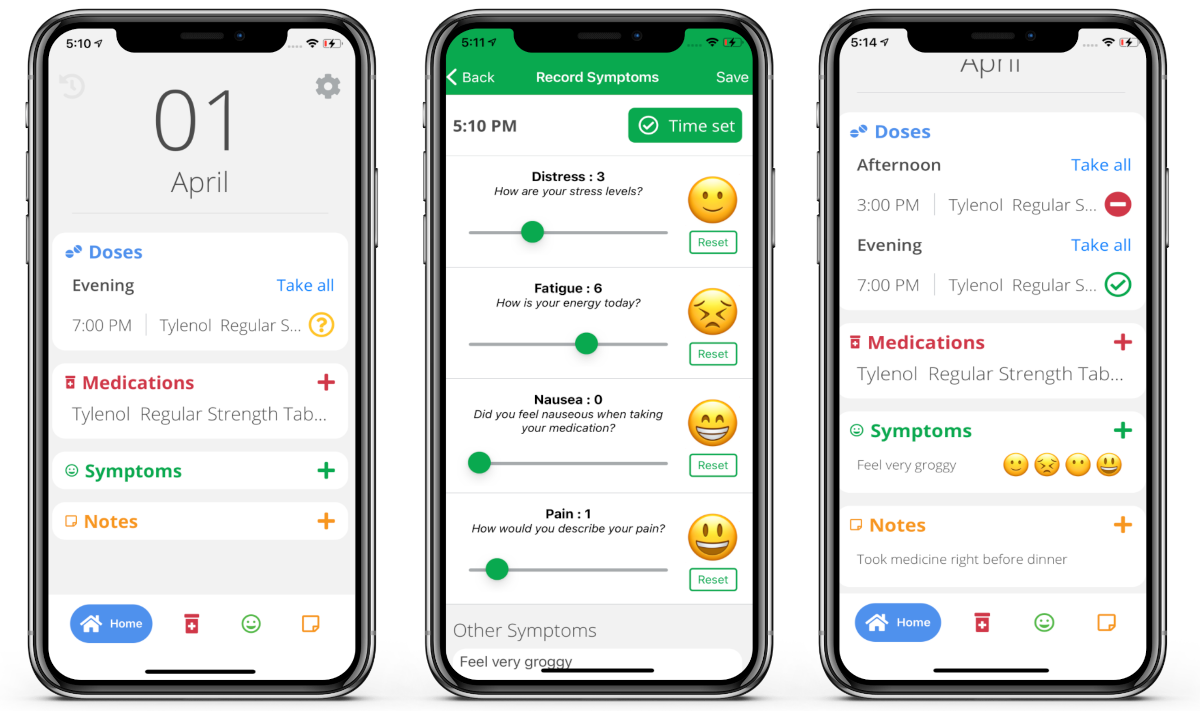
BMT4me app wireframes.

BMT4me (Figure 1) is a digital health intervention designed to aid in medication management, optimize adherence, and track symptoms or side effects in real time. The app reminds users of medication doses, records the time medication is taken, and asks reasons for missed doses through pop-up notifications. Medications can be typed in the app by the user or entered using an image-to-text option, which converts the medication label into text. As symptom severity can affect adherence and inform the need for medical intervention, it is illustrated by both corresponding emojis and numbers on a 1-to-10 scale to aid understanding and for convenient entry. Additional features include a notes page for recording any details of care to communicate with the provider and the ability to upload pictures. The app can be downloaded to both iOS and Android devices. Currently, availability is limited to the English language, and no clinical data is shared with the medical team during the research study.

### Research Aim and Rationale for Mixed Methods Methodology

The overall aim of this mixed methods study is to design and test a theory-driven mHealth app that can virtually assist caregivers of children post HSCT in improving medication adherence, tracking symptoms, and recording pertinent medical information via the journal feature. This app is different from other medication adherence apps available to patients with cancer in several ways: (1) features (eg, reminders, engaging emojis) in the app are designed to support medication adherence by applying BE principles to provide reminders and tailored notifications for caregivers; (2) it is specific to children after an HSCT; and (3) it provides more than medication reminders, with the design encouraging caregivers to engage with the app via symptom tracking and a journal feature that allows uploading of pictures.

## Methods

### Overall Study Design

**Figure 2:**
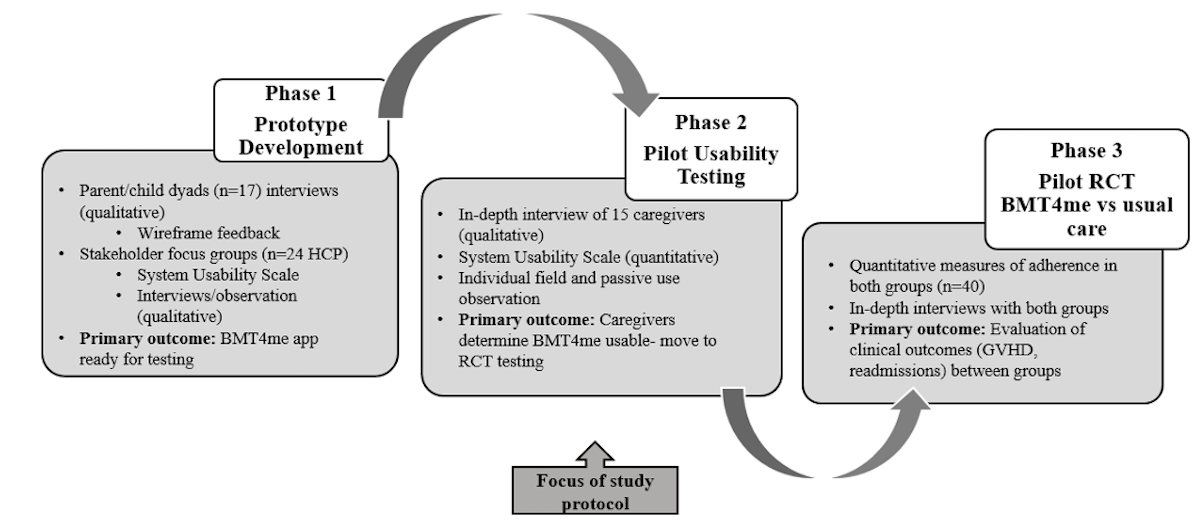
Multiphase mixed methods app design of BMT4me. GVHD: graft vs host disease; HCP: health care practitioner; RCT: randomized controlled trial.

The larger mixed methods mHealth app development study consists of 3 phases: (1) prototype development, (2) feasibility and usability, and (3) usability and clinical efficacy (Figure 2). This protocol corresponds to phase 2, feasibility and usability among 15 caregivers immediately upon discharge from the inpatient unit post HSCT. Inclusion criteria include (1) caregiver of child aged 2 to 18 years undergoing allogeneic transplant, (2) access to a smartphone (Android or iOS), and (3) English speaking. Findings from phase 1 informed phase 2, and thus findings from phase 2 will lead to further refinement prior to phase 3. In phase 3, the smartphone app will be compared to usual care in a pilot randomized controlled trial.

### Ethics Approval

All phases of the study have been approved by the Nationwide Children’s Hospital Institutional Review Board (approval number STUDY00000910).

### Phase 2 Study Design

**Figure 3:**
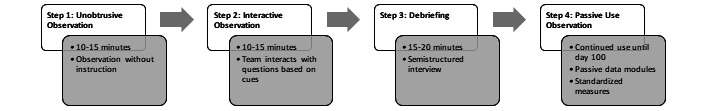
A stepped approach to usability testing with caregivers.

This is a convergent mixed methods design focused on analysis of quantitative and qualitative data simultaneously collected to obtain feedback on the adherence mHealth app interface, usability, and flow and content of the software. Usability testing is the principal means of determining if the system meets its intended purpose [31]. Purposive sampling will be used to identify caregivers of children across the spectrum of age, sex, and diagnoses of pediatric patients treated with HSCT. Caregivers will be evaluated for engagement and understanding of the app in the following steps (Figure 3):

- *Step 1. Unobtrusive observation to measure the intuitiveness of the interface (10-15 minutes).* Initially, caregivers will be briefly introduced to the program and asked to start interacting with the adherence mHealth app. The design team will not intervene or provide instructions and will observe and record participants’ progress.
- *Step 2. Interactive observation (10-15 minutes).* The design team will begin to interrupt individual participants during the interaction by posing questions regarding observed cues that imply success or delay in program use. Caregivers will be encouraged to verbalize their criticisms of the mHealth app. Since discussion will be based on evolving patterns of interaction, pre-prescribed questions are not possible.
- *Step 3. Debriefing (15-20 minutes).* Caregivers will be asked to reflect on their experience with the app and its interface and contents, and to indicate their opinions regarding the strengths and weaknesses of the app. These evaluations will be used to establish criteria for refining the content and interactive app features for future development.
- *Step 4. Passive use observation.* Caregivers will be asked to continue using the mHealth app on their own device until day 100 post HSCT or weaning of the immunosuppressant, whichever occurs first. Passive data modules will be added to the app to capture phone activity (screen on and off times) and will log the participants’ use of the app (eg, time, date, duration of use). Changes in app use and phone activity will be descriptively analyzed and plotted. Subjective measures and standard-of-care immunosuppression serum assays will be recorded weekly. Such passive observational effort of app use and adherence, with a mixed method approach, will be one of the first to be reported in an HSCT population.

At the conclusion, caregivers will complete a 15-minute semistructured interview related to participation in the intervention, as well as the System Usability Scale [32]. Open-ended feedback related to suggestions for further app development will undergo qualitative analysis.

### Data Collection

Trained research assistants will recruit and onboard eligible participants. The participants will sign written consent forms. The research assistant will then conduct one-on-one audio-recorded sessions, which will include a facilitator and a note taker. A project scientist may also participate. The measures listed below will be collected either electronically or in paper-and-pencil form from each caregiver. All participants will receive US $25 at enrollment and US $25 at the conclusion of the study after the exit interview and measures are complete.

#### The BMT4me App

The BMT4me app will collect daily data on medication-taking (taken or not taken), the time medication was taken, barriers or reasons for missed doses, symptoms, and any notes the caregiver completes. Passive use data on phone activity and app use will be collected.

#### Demographic Data Form

The caregiver will report on basic background characteristics, including parent and child age, sex, race, ethnicity, education level, and family income.

#### Medication Adherence Measure

The Medication Adherence Measure (MAM) is a semistructured interview specific to pediatrics, conducted with the parent, to obtain an individual score in each module. The score is represented as percentages of the number of required doses. A total summary score can be calculated across all medications as well separately. This allows for quantification of the degree of adherence on a continuum. MAM has demonstrated adequate convergent validity with MEMS (medication event monitoring system) Caps (*r*=−0.40, *P*<.05) [33]. MEMS Caps are electronic monitors that objectively measure adherence. They contain microelectronic circuits that date and time-stamp each instance the container is opened for a dose of medication. Data from the MEMS Caps will be downloaded using cloud- or computer-based software at each study visit [34,35].

#### System Usability Scale

The System Usability Scale is a 10-item questionnaire routinely used to evaluate the functionality and acceptability of mHealth apps [32,36]. Items are rated on a 5-point scale and scores range from 0 to 100 [36]. Reliability (0.91) and validity (0.81 correlation with a 7-point scale of “user-friendliness”) have been well established [37]. A score of >68% is considered above average [36].

#### Caregiver Satisfaction

Satisfaction will be assessed via semistructured interviews and electronic surveys with caregivers. Caregivers will be asked for feedback regarding participation in the intervention, benefits, burden, barriers, suggested modifications, and overall satisfaction. Suggested modifications to the app and advice to the health care team will also be solicited.

#### Medication Level Variability Index

The medication level variability index is the calculation of the standard deviation of serum assays of immunosuppressants that has been shown to correlate with adherence and clinical outcomes in the solid organ transplant population [38]. Immunosuppressant serum assays are collected weekly during the acute phase. The degree of variation among levels will also be calculated.

### Data Analysis

All interviews will be transcribed verbatim. During qualitative analysis, investigators will use an iterative process of reading, summarizing, and rereading data. The qualitative team is composed of 3 individuals with formal research training in psychology, behavioral health, and nursing. The interview transcripts will be organized and coded using NVIVO software (QSR International). In-vivo coding will occur as new themes emerge from the data. The data will be triangulated by comparing and integrating quantitative survey data with qualitative findings.

Quantitative data will be summarized with descriptive statistics (frequencies, means, and SDs). During the passive use observation period, passive data modules will capture phone activity and caregivers’ application use (eg, time, date, duration of use). Descriptive statistics will be used to analyze phone activity. Correlation analysis will be used to investigate use behavior over time. Acceptability will be assessed by averaging total scores from the System Usability Scale. Consistent with the literature [36], scores >68% on the System Usability Scale will be considered acceptable. The proportion of participants that enroll and complete the study will be examined to assess study feasibility.

The nature of the study is exploratory rather than confirmatory. Thus, the objective of the analysis is effect size estimation, rather than formal hypothesis testing. Threats to power (eg, participant attrition due to patient death, early taper of immunosuppressant medication) are not a primary concern. We will compute pre-post effect sizes (eg, a standardized mean difference) to inform the future randomized controlled trial (phase 3) assessing the efficacy of the intervention.

## Results

Enrollment of caregivers of children post HSCT from the free-standing Midwestern Children’s Hospital began in September 2021 and is ongoing. Findings from phase 2 will be used to inform further development and refinement of the app for testing in the pilot randomized controlled trial (phase 3) anticipated to begin in fall 2022. We anticipate high usability scores and a better understanding of unique features within the app that are needed for HSCT families post transplant. To date, usability scores among enrolled participants are greater than 70%. Feedback from qualitative interviews is being used to further adapt the app by adding specific weekly logs, call provider options, and voice to text. The results of this study will be disseminated through presentations at scientific conferences and publication in peer-reviewed journals.

## Discussion

To our knowledge, this is one of the first studies to use a systematic, phased approach to the development of a digital health intervention and evaluation of usability and feasibility to improve outcomes in pediatric HSCT. From this study, we anticipate high usability scores and a better understanding of unique features within the app that are needed for HSCT families post transplant. The long-term goal of this research is to develop novel digital interventions to increase adherence and ultimately improve clinical outcomes for these high-risk children. In addition, there is potential to adapt our work for children with other chronic conditions.

The strengths of the mixed methods approach and proposed digital health intervention include constructs from behavioral health and BE theory that drive data collection and analysis, as well as the use of multiple methods of data collection and multiple data sources to gain a comprehensive understanding of patient needs. The development of the intervention has undergone multiple iterations based on feedback from multiple stakeholders, including pediatric patients, caregivers, physicians, and nurses, each offering unique and important perspectives.

Potential limitations of the study include the use of 1 pilot recruitment site; however, there is a diverse group of patients requiring HSCT at this institution. A small number of patient caregivers will be recruited, and a larger user population could yield additional data. At this time, only English-speaking caregivers are eligible; opportunities to increase diversity by adding additional languages are planned in future iterations.

This protocol describes the second phase of a multiphase, theory-driven digital health app intervention to improve adherence and symptom tracking in children post HSCT. The study findings will provide important knowledge regarding the feasibility of testing in a larger randomized controlled trial. The goal of the entire project is to improve adherence and clinical outcomes in children post HCST, which may have important implications for improving adherence in other clinical populations.

## Appendix

Multimedia Appendix 1 Peer-review report by the National Institute of Nursing Research Initial Review Group (NRRC) (National Institutes of Health, USA).

## Abbreviations

BE: behavioral economics
HSCT: hematopoietic stem cell transplant
MAM: Medication Adherence Measure
MEMS: medication event monitoring system
mHealth: mobile health
