# Supplementary material for: An mHealth App to Promote Adherence to Immunosuppressant Medication and Track Symptoms in Children After Hematopoietic Stem Cell Transplant: Protocol for a Mixed Methods Usability Study
Source: JMIR Res Protoc. 2022 Jul 21;11(7):e39098. doi: 10.2196/39098 (PMC9353681; doi:10.2196/39098)
Supplement: Multimedia Appendix 1 [file resprot_v11i7e39098_app1.pdf]

SUMMARY STATEMENT
(Privileged Communication)

PROGRAM CONTACT:
MARTHA MATOCHA
301-594-2775
matocham@mail.nih.gov

Release Date: 07/20/2020
Revised Date:

Application Number: 1 K99 NR019115-01A1
Formerly: 1K99NR019115-01

Principal Investigator

SKEENS, MICAH

Applicant Organization: RESEARCH INST NATIONWIDE CHILDREN'S HOSP

Review Group: NRRC (78)
National Institute of Nursing Research Initial Review Group

Meeting Date: 06/25/2020
Council: OCT 2020
Requested Start: 10/01/2020
RFA/PA: PA19-129
PCC: AXZMM

Project Title: Improving adherence through mHealth for pediatric hematopoietic stem cell transplant patients
SRG Action: Impact Score:26
Next Steps: Visit https://grants.nih.gov/grants/next_steps.htm
Human Subjects: 30-Human subjects involved - Certified, no SRG concerns
Animal Subjects: 10-No live vertebrate animals involved for competing appl.
Gender: 1A-Both genders, scientifically acceptable
Minority: 1A-Minorities and non-minorities, scientifically acceptable
Age: 2U-Only Children, scientifically unacceptable

Project Year	Direct Costs Requested	Estimated Total Cost
1	82,302	83,494
2	82,820	84,019
3	249,000	252,606
4	249,000	252,606
5	249,000	252,606
TOTAL	912,122	925,332

ADMINISTRATIVE BUDGET NOTE: The budget shown is the requested budget and has not been adjusted to reflect any recommendations made by reviewers. If an award is planned, the costs will be calculated by Institute grants management staff based on the recommendations outlined below in the COMMITTEE BUDGET RECOMMENDATIONS section.

1K99NR019115-01A1 Skeens, Micah

INCLUSION ACROSS THE LIFESPAN UNACCEPTABLE

RESUME AND SUMMARY OF DISCUSSION: This is a resubmitted application for a Pathway to Independence Career Development Award from Dr. Micah Skeens, a postdoctoral fellow at the Research Institute at Nationwide Children's Hospital (NCH), seeking to identify effective strategies to increase medication adherence for children undergoing hematopoietic stem cell transplant (HSCT). Her long-term goal is to develop family-centered interventions to improve outcomes in this high-risk population During the K99 phase, in conjunction with training in mHealth Interventions, randomized controlled trials (RCT) and adherence, the candidate will conduct a mixed methods study to develop and pilot an iPhone-based medication reminder app geared toward parents for improving adherence to immunosuppressants in the acute outpatient post-transplant period. The R00 phase will assess acceptability of the app and feasibility for conducting a pilot RCT; initial efficacy of the app-based intervention for improving adherence relative to usual care will also be determined in support of a future R01 application. The well-prepared candidate with extensive clinical experience remains a strength of the resubmission, as do the well-matched team of experienced mentors, collaborators, and consultants and clear institutional support for the candidate's success. This resubmission is somewhat responsive to previous reviews by improving the Career Development Plan (CDP) through providing more specifics about K99 to R00 transition; adding an mHealth course and adherence conferences to bolster training in app design; and increasing interactions with Dr. Lin, the Chief Research Information Officer for NCH. The Research Plan now includes clinical outcomes in the specific aims; fidelity assessment and monitoring in the R00 phase; greater focus by removing the provider piece; and additional details, clarification, and attention to discrepancies between specific aims and approach. Dr. Skeens also added several publications and presentations; received three additional grants for app development; increased opportunities for face-to-face and virtual meetings with mentors; and specified how she will leverage the numerous resources available at NCH in support of the proposed career development and research activities. However, some previous concerns remain including insufficient detail about app design and development; inadequate training in RCTs; and potentially small number of available participants. Reviewers also note that while an app for this population is novel, the function is largely to provide education and misses opportunities to include interactive features (i.e., chat rooms, adherence reports) and obtain detailed information about app usage; further, mixed methods are limited and it is unclear how unobtrusive monitoring will be incorporated. Overall, reviewers appreciate the improvements in this resubmission and agree that this proposed research and training will increase the likelihood for Dr. Skeens to develop as an independent nurse-scientist; however, some weaknesses in the Career Development and Research Plans reduce the likely overall impact of this application which is ranked as outstanding for its scientific and technical merit.

DESCRIPTION (provided by applicant): An estimated 50-80% of all pediatric patients are non-adherent.3-6 Extensive pediatric adherence research exists in solid organ transplant, cystic fibrosis, diabetes, and other chronic illnesses. However, there are only three known studies in children with HSCT, and all report suboptimal adherence rates (52-78%) that worsen over time.7-9 Additionally, in pilot study, 70% of HSCT providers reported adherence as a major concern for their outpatient pediatric HSCT recipients.10 Currently, no HSCT specific interventions exist to improve adherence and clinical outcomes. There is a critical need to evaluate novel mHealth approaches to improve adherence and clinical outcomes among children receiving hematopoietic stem cell transplant (HSCT) to prevent morbidity and mortality. The rationale is that a mHealth app designed to send medication reminders to caregivers will increase adherence to immunosuppressant medication, thereby potentially reducing GVHD, readmissions, and mortality. The app will also obtain reasons for non-adherence adding to our knowledge of barriers to adherence in this population. This K99/R00 application describes the background and experience of the applicant, Micah A. Skeens PhD, RN, and her plan to acquire the knowledge and training necessary to become a leading independent clinical investigator in adherence

and mHealth interventions that improve clinical outcomes for children undergoing hematopoietic stem cell transplant (HSCT). The overall goal during the K99 is to gain advanced research training in mHealth interventions, adherence and conducting randomized controlled trials (RCTs) to develop and test a mHealth application for adherence of pediatric HSCT patients in the acute phase post-transplant. Using a behavioral economics approach, the goal of this new mHealth application will be to improve adherence and ultimately clinical outcomes as well as collect information regarding barriers. The research specific aim during the K99 phase is to develop an adherence mHealth application using a mixed methods approach and to evaluate the usability and acceptability of the mHealth application for pediatric HSCT patients in the acute outpatient phase post-transplant. To meet the K99 training objectives, a comprehensive training plan has been developed in concert with an internationally recognized interdisciplinary mentorship team of senior research experts. The overall goal during the R00 phase is to conduct a pilot RCT with 40 caregivers to assess acceptability, feasibility and potential efficacy of the newly developed mHealth app. The research specific aims during the R00 phase are to: 1) evaluate the acceptability of a newly developed mHealth app and the feasibility of enrolling and retaining 40 caregivers of children in the acute outpatient phase post-HSCT and 2) evaluate the potential efficacy of the mHealth app on adherence to immunosuppressants in children who have been discharged home during the acute phase post-HSCT. The results will directly support an R-01 application to conduct a multi-site RCT efficacy trial of the mHealth application on adherence and clinical outcomes in children during the acute phase post-HSCT.

PUBLIC HEALTH RELEVANCE: In the United States, poor adherence accounts for up to 70% of all medication-related hospital admissions and an estimated 50-80% of pediatric patients are non-adherent. Behavioral economics (BE) suggests that small “nudges” or reminders can produce and sustain behavior change, thus with mobile health (mHealth) access nearly ubiquitous and an estimated 3.7 billion mHealth application downloads in 2017 alone,13 technology has great potential to improve adherence. Nurses could easily deliver these universal mHealth intervention approaches as a frontline approach to family education and care, addressing the critical need to improve adherence and clinical outcomes among children to ultimately prevent morbidity and mortality.

CRITIQUE 1

Candidate: 1
Career Development Plan/Career Goals /Plan to Provide Mentoring: 3
Research Plan: 2
Mentor(s), Co-Mentor(s), Consultant(s), Collaborator(s): 1
Environment and Institutional Commitment to the Candidate: 1

Overall Impact: Resubmission application from a post-doc fellow completing her first year at the Research Institute at Nationwide Children’s Hospital (NCH). The application has multiple strengths. The candidate has substantial clinical grounding, strong foundational training, and clinical trial experience that result in excellent potential for development as an independent clinician-scientist. The mentorship team is well-matched to the candidate’s research and development goals, and the career development plan includes a variety of valuable training activities. The proposed research is sufficiently justified and of appropriate scope for the candidate’s stage of development. Design, procedures, and analysis plans for both the K99 usability study and R00 pilot RCT are largely sound, with minor weaknesses that should be easily addressed by the candidate prior to implementation. NCH and the Ohio State University provide numerous resources that will support the candidate in successfully achieving her research goals and transition to independence. Some weaknesses persist from the previous submission, specifically (1) minimal description of how new knowledge in behavioral theory and mHealth interventions obtained during the K99 phase will be incorporated into app development, and (2) lack of hands on experiential training for conduct of multi-site behavioral intervention trials before the independent R00 phase. Overall, the application proposes valuable career development for a

candidate with high potential and good likelihood to transition to independence despite identified weaknesses.

1. Candidate

Strengths

- Good foundational training and relevant clinical trial experiences.
- 20 years of clinical experience in pediatric oncology.
- Excellent record of productivity for the candidate's career stage (20+ publications, 6 first authored).
- Has successfully competed for 3 foundation small grants that are supporting her current app development work.
- Referee letters indicate strong potential to become a productive independent clinician-scientist.
- Very good likelihood that the candidate can achieve an independent faculty position within the K99 period.
- Track record of collaborative trials with the Children's Oncology Group indicates the candidate has the ability to organize and manage the proposed clinical studies.

Weaknesses

- None.

2. Career Development Plan/Career Goals & Objectives

Strengths

- Builds on clinical trial and dissertation research examining adherence to immunosuppressants in pediatric HSCT patients.
- Addresses gaps in the candidate's training specific to behavioral health theories, mHealth interventions, and mixed methods research.
- Career development plan includes didactic and experiential learning activities and takes advantage of numerous professional development workshops and seminars available.
- K99 includes careful oversight with progress evaluations by mentors (quarterly) and a scholarship oversight committee (twice yearly) to gauge and support the candidate's transition to independence.
- Includes plans to search for a faculty position and identifies target institutions with large pediatric HSCT programs.

Weaknesses

- While the candidate makes a case that training is needed beyond her experience conducting cooperative group treatment trials, there is no plan for mentored experiential training in the implementation / conduct of "investigator-initiated multi-site behavioral clinical trials" before the independent R00 phase.
- While the addition of the NIH mHealth Institute in Year 1 is a substantial improvement to the career development plan, adherence conferences added to address noted weaknesses occur after the K99 phase.

- Minimal description of the app development process (K99 aim 1). How will new knowledge of behavioral health theory and mHealth obtained during the K99 training be incorporated into the app?
- Identifies 2-3 manuscripts per year as a benchmark, but specific topics are not described.

3. Research Plan

Strengths

- Good scientific basis, sound design and analysis plans for K99 usability and R00 feasibility and acceptability pilot RCT.
- Development of the app prototype is already in progress with input from provider stakeholders.
- Identifies potential problems and reasonable alternative strategies.
- If the intervention is demonstrated as feasible and acceptable, the candidate will be well-positioned from a subsequent multi-site R01 efficacy trial.

Weaknesses

- Limited innovation – content is limited to medication reminders and dosing log. Are there plans to further develop self-management support for caregivers in future iterations?
- The primary outcome of the pilot RCT appears to change throughout the application (feasibility & acceptability vs preliminary efficacy/adherence). Limitations in using a pilot study to estimate effect size should be noted (for example, see https://www.nccih.nih.gov/grants/pilot-studies-common-uses-and-misuses).
- The power analysis section notes plan to use a different control condition in the subsequent R01 (enhanced usual care or attention control). It would be valuable to assess feasibility of the planned control group in the R00 pilot study.
- Excluding the control group from interviews at the end of the study misses an opportunity to assess study feasibility and capture information about other steps taken to facilitate adherence when caregivers learn they will not receive the medication reminder app.
- No analysis plans for comparison of Medication Level Variability Index (MLVI) to other adherence measures (exploratory aim).
- If the caregiver is the person using the app and administering the medication, what is the rationale for excluding children with developmental delay?

4. Mentor(s), Co-Mentor(s), Consultant(s), Collaborator(s)

Strengths

- Primary mentor (Dr. Cynthia Gerhardt) is a pediatric psychologist with expertise in behavioral theories and mixed methods research in children with cancer. Has ongoing behavioral intervention research that could support the candidate’s training. Good track record of funding & dissemination.
- Two distance co-mentors provide expertise related to adherence in pediatric oncology: a senior nurse scientist at UAB (Landier) and a pediatric psychologist at Cincinnati Children’s Hospital (Pai).
- Three collaborators at OSU bring complementary expertise in medical informatics and mHealth patient engagement (Lin), biostatistics (Rausch) and behavioral economics (Stevens).

- A pediatric psychology consultant at Mount Sinai School of Medicine (Dr. Shemesh) will provide guidance in use and interpretation of the adherence biomarker, Medication Level Variability Index.
- All members of the mentorship team have active research; several have extensive experience mentoring trainees and junior faculty.
- There is a clear plan for frequent individual and quarterly group meetings; letters of support commit to the planned mentorship and meetings.

Weaknesses

- None.

5. Environment and Institutional Commitment to the Candidate

Strengths

- Appropriate research facilities, training resources, and professional development opportunities at the Research Institute and Patient-Centered Pediatric Research Program at Nationwide Children's Hospital plus supplementary opportunities through the Center for Biobehavioral Health, OSU Center for Clinical and Translational Science, and Cincinnati's T32 training program in adherence.
- NCH indicates at least the minimum required 75% protected time, funding to support research expenses beyond the K99/R00 award, and consideration of a faculty position following completion of the K99 phase.

Weaknesses

- None.

Study Timeline

Strengths

- Feasible time frame presuming the candidate stays at Nationwide Children's Hospital or transitions to an institution with a similarly sized HSCT program.
- Adequate recruitment and retention plans.

Weaknesses

- None.

Protections for Human Subjects: Acceptable Risks and Adequate Protections

- Risks to both the caregiver and child are addressed in human subjects' protection.

Data and Safety Monitoring Plan (Applicable for Clinical Trials Only): Acceptable

Inclusion Plans

- Sex/Gender: Distribution justified scientifically
- Race/Ethnicity: Distribution justified scientifically
- Inclusion/Exclusion Based on Age: Distribution not justified scientifically
- The age range of 2-12 years is for the children only; does not include the caregivers.

Vertebrate Animals: Not Applicable (No Vertebrate Animals)

Biohazards: Not Applicable (No Biohazards)

Resubmission: Responsive to weaknesses previously noted in the research strategy, plan for K99 to R00 transition, and the candidate's use of resources at NCH. However, limitations noted in the app design phase / development process and RCT training remain concerns.

Training in the Responsible Conduct of Research: Acceptable

Comments on Format (Required):

- Includes online and face-to-face, lecture and discussion.

Comments on Subject Matter (Required):

- Includes NIH recommended subject matter.

Comments on Faculty Participation (Required; not applicable for mid- and senior-career awards):

- Mentors are involved in informal RCR training.

Comments on Duration (Required):

- > 8 hours.

Comments on Frequency (Required):

- Ongoing throughout training.

Resource Sharing Plans: Not Applicable (No Relevant Resources)

Budget and Period of Support: Recommend as Requested

CRITIQUE 2

Candidate: 1
Career Development Plan/Career Goals /Plan to Provide Mentoring: 2
Research Plan: 3
Mentor(s), Co-Mentor(s), Consultant(s), Collaborator(s): 3
Environment and Institutional Commitment to the Candidate: 1

Overall Impact: This is a resubmission of a K99/R00 application from Dr. Skeens, a post-doctoral fellow at Wexner Research Institute, Nationwide Children's Hospital (RINCH). Dr. Skeens has 20 years of clinical experience in pediatric oncology/transplant units. Her proposed research builds on her dissertation and is on strategies to increase medication adherence for children undergoing hematopoietic stem cell transplant (HSCT). She is an excellent candidate, with a number of publications and recent foundation grant funding. Overall, it appears Dr. Skeens was responsive to the majority of the previous reviewer's comments. For her training plan, some areas that might need to be included are identified. For the proposed research, there are still some questions, for example, about the proposed app and data collected. She has a large mentoring team, with needed skills, although as noted before a team of 7 seems large, and the lack of onsite nurse mentor is noted. RINCH appears to have the resources needed for Dr. Skeens' success.

1. Candidate

Strengths

- Dr. Skeens is currently a post-doctoral fellow at Wexner Research Institute, Nationwide Children's Hospital (RINCH), Columbus OH after completing a PhD in Nursing at Vanderbilt in 2018.
- Clinical experience as an RN for 4 years and then as a NP for 12 years in the bone marrow transplant (BMT) unit and 5 years on the Embryonal Tumor Team at Nationwide Children's Hospital in Columbus OH.
- Research focus area is caring for children with cancer and blood disorders and their families. Dissertation was on adherence to immunosuppressants in pediatric stem cell transplant patients. Proposed study is on strategies to increase medication adherence for children undergoing hematopoietic stem cell transplant (HSCT).
- Member of a number of professional organizations such as Children's Oncology Group, Bone Marrow foundation, Association of Pediatric Hematology Oncology Nurses.
- Honors include Nursing Leadership Award for Children's Oncology Group.
- 16 publications from 2012-present, one dissertation paper published, second under revision.
- Currently PI on a Daisy Foundation grant and Oncology Nurses Foundation.
- Dr. Skeens provided a letter listing additional publications since grant submission, one from 2019 and 4 since March 2020.
- Letters of support for Dr. Skeens are extremely strong, including one from the Chief Scientific Officer at RINCH.

Weaknesses

- Minor confusion over current funding. A third grant is mentioned and on the biosketch it appears to be an America Nurses Credentialing Center grant, though no dates listed. Later there is a statement about an OSU CTSS grant, but not listed on the biosketch.

2. Career Development Plan/Career Goals & Objectives

Strengths

- Includes 4 training objectives that focus on 1) behavioral theory/adherence, 2) mHealth technology (apps), 3) mixed methods, 4) multi-site RCTs.
- Has added training requested by previous reviewers.

Weaknesses

- Would expect inclusion of more conferences to attend across all 5 years.
- Since the app is developed on behavioral economics theory thought there might be training focused on behavioral economics and adherence.
- In order to develop skills in app development, may need more than just workshops. For example, may want to take a course on Human Factors which are often offered in engineering.
- Not clear what training in RCTs will occur.
- NIH Methodologies Boot Camp, listed for mixed methods, is not always about mixed methods. (This year it's on AI). Might explore a course at OSU CON on mixed methods.

3. Research Plan

Strengths

- K99 is to develop and explore usability and acceptability of an app targeting adherence to immunosuppressant therapy in children post HSCT.
- R00 is also to test acceptability of the app and to evaluate the efficacy of the app.
- Provides an overview that highlights lack of research in adherence in this group of children; notes 52-73% adherence rates.

Weaknesses

- In background, it states that with data obtained from the app, families identified at greater risk for non-adherence could be referred. It isn't clear what the data is that will be used from the app to identify non-adherence. There may be an opportunity to develop a predictive model, but the app appears to only remind the caregiver. Is it the barrier component that might be predictive? Would be helpful to describe how barrier data is collected and coded. It doesn't appear that the app will have med adherence measures as part of it or MLVI; these appear to be separate.
- The app will include reminders, track adherence (not clear if this means parents document that they have given the medication), and capture barriers. Innovation highlights measure of adherence, but these appear to be separate measures and not part of the app.
- App is in development, would be helpful to know the role of Dr. Skeens in this process beyond providing content.
- For the Medication Adherence Measure, it would be helpful to know the number of items, psychometrics, and how long this takes to complete. It is noted in the potential problems that weekly assessments may be burdensome.
- In the R00 phase, assume the table of study measures are the same for both groups, except for the System Usability Scale and the Caregiver Satisfaction which seem to be only for the app group, although not clear.
- Under potential problems, there is a reference to recruitment of non-adherent families being difficult. It doesn't appear there was a plan to enroll nonadherent families, just families in general.

4. Mentor(s), Co-Mentor(s), Consultant(s), Collaborator(s)

Strengths

- Dr. Skeens has an interdisciplinary team with a range of expertise.
- Mentor is Cynthia Gerhardt, PhD, Professor of Psychology at Ohio State University and in Pediatrics at RINCH and Director of the Center for Biobehavioral Health at RINCH. A pediatric psychologist with a research focus on childhood cancer. Research now focused on developing interventions. Appears to have expertise to mentor Dr. Skeens. Will meet weekly during training
- Co-mentors: Wendy Landier, PhD, RN, Professor in Pediatrics at U of Alabama, research on childhood cancer survivors, Chair of Children Oncology Group-Nursing. Ahna Pai, PhD, Professor, Behavioral Medicine and Pediatrics, U of Cincinnati and Cincinnati Children's, expertise in adherence in stem cell patients and electronic behavioral interventions.
- Collaborators: Joseph Rausch, PhD, biostats. Jack Stevens, PhD, Associate Professor, Pediatrics Ohio State and Psychologist, Sleep Disorders Clinic, NCH, Behavioral economics

expert, Simon Lin, MD, MBA, Chief Information Officer at RINCH, has developed health information technology, apps.

- Consultant: Eyal Shemesh, MD, Director, Pediatric Clinical Trials Office, Mt Sinai.
- Scholarship Oversight Committee will provide direction to the mentoring team. Includes mentors, external nurse oncology researcher, a local MD (Cripe) in pediatric oncology and another psychologist.

Weaknesses

- As noted, before, a mentoring team of 7 seems excessive. For example, both Drs. Gerhardt and Pai seem to have similar areas of expertise for mentoring Dr. Skeens.
- Not clear what the difference is among the groups: Collaborators vs consultant; mentoring team vs oversight committee.
- As noted, only one nurse scientist who is not at RINCH seems to be a concern for career development during the K99 component, although Dr. Landier is a leading expert in pediatric adherence research and nursing.
- Only meeting monthly with most of the mentoring team. May need more time.

5. Environment and Institutional Commitment to the Candidate

Strengths

- Research Institute, Nationwide Children's Hospital (RINCH) appears to have the resources needed for his project.
- Per mentor Gerhardt, Dr. Skeens will have at least 75% release time (actually 80%) to devote to the K99 and the R00, although R00 component in theory may not occur at RINCH.

Weaknesses

- Not clear how many children have a HSCT each year at NCH. A previous reviewer noted 50 per year. If this is accurate, although number in each phase are decreased, may still be a concern. However, R00 may not occur at NCH.

Study Timeline

Strengths

- A table provides the overview of the timeline.

Weaknesses

- Per note of previous reviewer, there may still be concerns about enough potential subjects for the two studies.

Protections for Human Subjects: Acceptable Risks and Adequate Protections

- Caregivers (primarily parents) are subjects in both K99 and R00. However, data will be obtained about children, so they also appear to be subjects.

Data and Safety Monitoring Plan (Applicable for Clinical Trials Only): Acceptable

- The R00 is a clinical trial. Dr. Skeens outlines a DSMP and will defer to the IRB on the potential need for a DSMB.

Inclusion Plans

- Sex/Gender: Distribution justified scientifically
- Race/Ethnicity: Distribution justified scientifically
- Inclusion/Exclusion Based on Age: Distribution not justified scientifically
- Includes caregivers of children 2-12 and those children.

Vertebrate Animals: Not Applicable (No Vertebrate Animals)

Biohazards: Not Applicable (No Biohazards)

Resubmission: Overall, Dr. Skeens appears to have responded to much of previous reviewers' concerns.

Training in the Responsible Conduct of Research: Acceptable

Comments on Format (Required):

- Online, small groups, in-person discussions.

Comments on Subject Matter (Required):

- Includes NIH required topics.

Comments on Faculty Participation (Required; not applicable for mid- and senior-career awards):

- Members of mentoring team are included.

Comments on Duration (Required):

- At least 8 hours per week.

Comments on Frequency (Required):

- Weekly throughout.

Resource Sharing Plans: Not Applicable (No Relevant Resources)

Budget and Period of Support: Recommend as Requested

CRITIQUE 3

Candidate: 1
Career Development Plan/Career Goals /Plan to Provide Mentoring: 2
Research Plan: 2
Mentor(s), Co-Mentor(s), Consultant(s), Collaborator(s): 1
Environment and Institutional Commitment to the Candidate: 1

Overall Impact: This is a K99/R00 resubmission by Dr. Skeens. The PI has covered the majority of the reviewers' recommendations. While this is a very ambitious proposal that will require rigor in maintaining the research timeline, the PI has proposed a convincing argument and support of this proposal. This is a very strong proposal from the well-qualified PI with strong clinical background in pediatric oncology. The team is highly skilled in their respective areas that will aptly support the PI in

the needed training activities and research plan. Overall, it was a pleasure to read Dr. Skeens' resubmission.

1. Candidate

Strengths

- Post-doctoral fellowship at the Abigail Wexner Research Institute at Nationwide Children's Hospital in Columbus, OH.
- Awarded – Vanderbilt Centennial Fellow and 2 doctoral scholarships from Oncology Nursing Society and American Cancer Society.
- 20 years clinical experience in pediatric oncology and HSCT.
- While in doctoral studies she published 9 manuscripts; 3 as first author.
- She is site PI for a multi-site study that is examining at the effectiveness and feasibility of 2 parent education discharge strategies for parents of children newly diagnosed with CA and is co-investigator on other institutional trials examining supportive care for pediatrics with HSCT and late effects in pediatric oncology.
- PhD in nursing science, clinical research track from Vanderbilt University.
- Independent study in qualitative research and investigated an adherence biomarker (MLV1) and association with post-HSCT.
- She participated in the Patient-Centered Pediatric Research Program for clinical research fellows at NCH.
- She has published 3 papers since last reviewed; given 5 presentations; and has 5 papers under review.
- She secured new funding from Oncology Nurses Foundation to develop an adherence mobile health app for pediatric stem cell transplant patients using focus groups of parents and health care providers; and in addition, two other sources of funding.
- Has established collaboration with Dr. Lin in the development of an mHealth app; attended NIH on MHealth Institute and 2 adherence conferences to enhance her learning objective training in app design; she has also been working with Dr. Gerhardt since last summer on the proposed app.
- To complete the R00 research she is planning on applying to: CHLA, Texas, Seattle Children's, and Cincinnati Children's.
- She aims to build a program of research to develop and evaluate interventions that improve adherence and ultimately clinical outcome.
- Since last summer, the PI has been working with Dr. Gerhardt.

Weaknesses

- None.

2. Career Development Plan/Career Goals & Objectives

Strengths

- The mentored K99 phase training has been designed to provide the PI with training in several areas that appropriate and well-justified for the candidate's current stage of scientific and

professional development and proposed research career goals. The stated training gaps are in health behavior theories for medication adherence; mixed method approaches and the assessment of adherence; the use of innovative eHealth/mHealth technologies in pediatric adherence; and advanced training in design, implementation and analysis of biobehavioral RCTs.

- The plan is to meet with Drs. Gerhardt, Landier and Pai will evaluate the progress on a quarterly basis which seems worthwhile and a goal to ensure the PI is progressing adequately and appropriate for guiding the applicant towards a successful transition to the independent phase of the award.
- Table 1 outlining the career development has been improved and clarifies any previous questions; very succinct.
- This is a very ambitious career development proposed which if executed correctly and rigorously will potentially contribute substantially to the scientific and professional development of the PI and facilitate her successful transition to independence.
- The timeline planned for transition to the independent phase of the award appropriate for the candidate's current stage of scientific and professional development is appropriate for the K99 phase of the award.
- PI attended to career training recommendations by the reviewers.

Weaknesses

- Training in RCTs was not addressed.
- App development was partially addressed in the resubmission but not adequately to understand what she did to develop the app; how was she involved; what parts did she develop and more of her training was needed in this area.

3. Research Plan

Strengths

- The proposed K99 phase research is highly significant and scientifically sound with a novel population of study.
- Adequate prior research that will serve as key support for the proposed research; the PI also added further documentation in this version of the proposal.
- The scientific and technical merits of her previous work leading to the K99 research is appropriate for developing the research skills outlined in the career development plan and appropriate for developing a successful R00 research program.
- The R00 phase research is clear, significant, scientifically sound and a logical extension of the K99 phase research.
- Fidelity measures were stated appropriately in the R00 phase research – the PI will monitor the ensure intervention fidelity; methodological procedures include fidelity processes as well.
- Study approach was also improved with clearly outlining the study sample, measures and procedures.
- Clinical outcome measures were addressed in R00 phase research.
- Potential problems and alternative strategies are adequately addressed.

Weaknesses

- Seems like the app is being developed now. Has the PI been involved in developing the app?
- Why is it that another type of reminder device is required for parents? Is there something more sophisticated that would be more useful? Is this device truly any more novel than another reminder device?

4. Mentor(s), Co-Mentor(s), Consultant(s), Collaborator(s)

Strengths

- Primary mentor, Dr. Cynthia Gerhardt, is a pediatric psychologist and has 20 years of conducting research; she is on 10 grants and 3 as PI and 1 MPI.
- The candidate has organized a team of co-mentors and collaborators: Landier is an expert in adherence in pediatric leukemia and conducts longitudinal, multi-site trials as well as qualitative and mixed methods; Dr. Pai is an expert in pediatric HSCT adherence and psychosocial adjustment as well as intervention development and the conduct of behavioral RCTs. The PIs collaborators include Dr. Lin will provide training on mHealth technologies; Stevens is an expert in the design and analysis of clinical trials; Dr. Shemesh is a consultant who provide consultation on the MLVI methodology and the development of multi-site RCTs for adherence.
- For each mentor, collaborator and consultant, meeting time have been provided and seem reasonable.
- Has identified members on the Scholarship Oversight Committee. Letter of support from Dr. Cripe.

Weaknesses

- While the team is quite large; how does the PI intend to manage such a large mentoring team? Is everyone necessary?

5. Environment and Institutional Commitment to the Candidate

Strengths

- Strong environment and an appropriate fit for the PI.
- PI enhanced this section of the proposal and addressed reviewers' concerns.
- Strong institutional commitment and letters of support from mentors, collaborators, consultant and from members of the Scientific Advisory Committee.

Weaknesses

- None.

Study Timeline

Strengths

- Ambitious; rigorous for K99 and R00.

Weaknesses

- None.

Protections for Human Subjects: Acceptable Risks and Adequate Protections

Data and Safety Monitoring Plan (Applicable for Clinical Trials Only): Acceptable

- Addressed children as human subjects although qualifying that no direct measures will be conducted with the children; appropriate training of staff and participants is outlined.

Inclusion Plans

- Sex/Gender: Distribution justified scientifically
- Race/Ethnicity: Distribution justified scientifically
- Inclusion/Exclusion Based on Age: Distribution not justified scientifically

Vertebrate Animals: Not Applicable (No Vertebrate Animals)

Biohazards: Not Applicable (No Biohazards)

Resubmission: PI has addressed major concerns from the reviewers; perhaps, the only item that seems somewhat concerning may be the timeline for recruitment in R00, however, there is confidence since the PI with mentors will entertain necessary alternatives so that the PI does not get delayed.

Training in the Responsible Conduct of Research: Acceptable

Comments on Format (Required):

- In person, online, lecture and mentorship.

Comments on Subject Matter (Required):

- NIH topics.

Comments on Faculty Participation (Required; not applicable for mid- and senior-career awards):

- Mentors will participate in informational RCR discussions.

Comments on Duration (Required):

- 6-8 hours.

Comments on Frequency (Required):

- CITI once during award period; RCR certification annually; didactic sessions weekly.

Resource Sharing Plans: Not Applicable (No Relevant Resources)

Budget and Period of Support: Recommend as Requested

THE FOLLOWING SECTIONS WERE PREPARED BY THE SCIENTIFIC REVIEW OFFICER TO SUMMARIZE THE OUTCOME OF DISCUSSIONS OF THE REVIEW COMMITTEE, OR REVIEWERS' WRITTEN CRITIQUES, ON THE FOLLOWING ISSUES:

PROTECTION OF HUMAN SUBJECTS: ACCEPTABLE

DATA AND SAFETY MONITORING PLAN: ACCEPTABLE

INCLUSION OF WOMEN PLAN: ACCEPTABLE

INCLUSION OF MINORITIES PLAN: ACCEPTABLE

INCLUSION ACROSS THE LIFESPAN: UNACCEPTABLE
The age range for inclusion is 2-12 years; however, this does not include the caregiver participants.

TRAINING IN THE RESPONSIBLE CONDUCT OF RESEARCH: ACCEPTABLE

COMMITTEE BUDGET RECOMMENDATIONS: The budget was recommended as requested.

Footnotes for 1 K99 NR019115-01A1; PI Name: Skeens, Micah

NIH has modified its policy regarding the receipt of resubmissions (amended applications).See Guide Notice NOT-OD-18-197 at https://grants.nih.gov/grants/guide/notice-files/NOT-OD-18-197.html. The impact/priority score is calculated after discussion of an application by averaging the overall scores (1-9) given by all voting reviewers on the committee and multiplying by 10. The criterion scores are submitted prior to the meeting by the individual reviewers assigned to an application, and are not discussed specifically at the review meeting or calculated into the overall impact score. Some applications also receive a percentile ranking. For details on the review process, see http://grants.nih.gov/grants/peer_review_process.htm#scoring.

MEETING ROSTER

National Institute of Nursing Research Initial Review Group
NATIONAL INSTITUTE OF NURSING RESEARCH
NRRC (78)
06/25/2020 - 06/26/2020

Notice of NIH Policy to All Applicants: Meeting rosters are provided for information purposes only. Applicant investigators and institutional officials must not communicate directly with study section members about an application before or after the review. Failure to observe this policy will create a serious breach of integrity in the peer review process, and may lead to actions outlined in NOT-OD-14-073 at https://grants.nih.gov/grants/guide/notice-files/NOT-OD-14-073.html and NOT-OD-15-106 at https://grants.nih.gov/grants/guide/notice-files/NOT-OD-15-106.html, including removal of the application from immediate review.

CHAIRPERSON(S)

ALBERT, STEVEN M, PHD
PROFESSOR AND CHAIR
GRADUATE SCHOOL OF PUBLIC HEALTH
UNIVERSITY OF PITTSBURGH
PITTSBURGH, PA 15261

MEMBERS

AGOPIAN, A.J., PHD *
ASSOCIATE PROFESSOR
DIVISION OF EPIDEMIOLOGY
UNIVERSITY OF TEXAS HEALTH SCIENCE CENTER
HOUSTON, TX 77030

BLAUM, CAROLINE S, MD, BA, MS *
PROFESSOR
SCHOOL OF MEDICINE
NEW YORK UNIVERSITY
NEW YORK, NY 10016

CHRISTIAN, LISA MICHELLE, BA, BS, PHD, MA *
ASSOCIATE PROFESSOR
DEPARTMENT OF PSYCHIATRY & BEHAVIORAL HEALTH
THE OHIO STATE UNIVERSITY
COLUMBUS, OH 43210

CIOE, PATRICIA ANN, PHD *
ASSISTANT PROFESSOR
BEHAVIORAL AND SOCIAL SCIENCES
BROWN UNIVERSITY
PROVIDENCE, RI 02912

CONG, XIAOMEI SOPHIA, PHD, RN, FAAN
PROFESSOR AND ASSOCIATE DEAN FOR RESEARCH AND SCHOLARSHIP
SCHOOL OF NURSING
UNIVERSITY OF CONNECTICUT
STORRS, CT 06269

CORWIN, ELIZABETH JEANNE, BS, PHD, MS, BSN *
PROFESSOR AND ASSOCIATE DEAN FOR RESEARCH
SCHOOL OF NURSING
COLUMBIA UNIVERSITY
NEW YORK, NY 10032

DEVON, HOLLI A, BSN, FAAN, MS, PHD *
PROFESSOR
SCHOOL OF NURSING
UNIVERSITY OF CALIFORNIA LOS ANGELES
LOS ANGELES, CA 90095

DOCHERTY, SHARRON L, PHD, RN, FAAN
ASSOCIATE PROFESSOR AND DIRECTOR
SCHOOL OF NURSING
DUKE UNIVERSITY
DURHAM, NC 27710

DORSEY, SUSAN G, PHD, RN, FAAN
PROFESSOR AND CHAIR
SCHOOL OF NURSING
UNIVERSITY OF MARYLAND
BALTIMORE , MD 21201

ELLINGTON, LEE A, PHD
PROFESSOR
HUNTSMAN CANCER INSTITUTE
COLLEGE OF NURSING
UNIVERSITY OF UTAH
SALT LAKE CITY, UT 84112

HAPP, MARY E, MSN, PHD, BSN, RN, FAAN *
PROFESSOR AND ASSOCIATE DEAN
SCHOOL OF NURSING
THE OHIO STATE UNIVERSITY
COLUMBUS, OH 43210

KAUFMANN, PETER G, MS, PHD *
PROFESSOR AND ASSOCIATE DEAN FOR RESEARCH AND INNOVATION
M. LOUISE FITZPATRICK COLLEGE OF NURSING
VILLANOVA UNIVERSITY
VILLANOVA, PA 19085

KELECHI, TERESA J, MSN, BSN, PHD *
PROFESSOR
COLLEGE OF NURSING
MEDICAL UNIVERSITY OF SOUTH CAROLINA
CHARLESTON, SC 29425

KWEKKEBOOM, KRISTINE L, PHD, RN, FAAN
PROFESSOR AND VICE CHAIR
SCHOOL OF NURSING
UNIVERSITY OF WISCONSIN-MADISON
MADISON, WI 53705

LEWIS, LISA M, PHD, RN, FAAN
ASSOCIATE PROFESSOR AND ASSISTANT DEAN
SCHOOL OF NURSING
UNIVERSITY OF PENNSYLVANIA
PHILADELPHIA, PA 19104

MCCARTHY, ANN MARIE, PHD, RN, FAAN
PROFESSOR AND ASSOCIATE DEAN
COLLEGE OF NURSING
THE UNIVERSITY OF IOWA
IOWA CITY, IA 52242

PORTILLO, CARMEN J, PHD, RN, FAAN *
PROFESSOR
SCHOOL OF NURSING
YALE UNIVERSITY
ORANGE, CT 06477

WEISS, SANDRA JEAN, PHD, DNSC, FAAN, RN *
PROFESSOR AND ESCHBACH ENDOWED CHAIR
SCHOOL OF NURSING
UNIVERSITY OF CALIFORNIA, SAN FRANCISCO
SAN FRANCISCO, CA 94143

WOO, MARY ANN, PHD, RN, FAAN
PROFESSOR OF NURSING
SCHOOL OF NURSING
UNIVERSITY OF CALIFORNIA, LOS ANGELES
LOS ANGELES, CA 90095

YEO, SEONAE, PHD *
PROFESSOR
SCHOOL OF NURSING
UNIVERSITY OF NORTH CAROLINA AT CHAPEL HILL
CHAPEL HILL, NC 27599

SCIENTIFIC REVIEW OFFICER

NORDSTROM, CHERYL, PHD
SCIENTIFIC REVIEW OFFICER
NATIONAL INSTITUTE OF NURSING RESEARCH
NATIONAL INSTITUTES OF HEALTH
6701 DEMOCRACY BLVD., SUITE 703H
BETHESDA, MD 20892

OTHER REVIEW STAFF

KELLY, MARY A
PROGRAM SPECIALIST
NATIONAL INSTITUTE OF NURSING RESEARCH
NATIONAL INSTITUTES OF HEALTH
BETHESDA, MD 20892

STOCKING, SHAWN
PROGRAM ANALYST
NATIONAL INSTITUTE OF NURSING RESEARCH
NATIONAL INSTITUTES OF HEALTH
BETHESDA, MD 20892

* Temporary Member. For grant applications, temporary members may participate in the entire meeting or may review only selected applications as needed.

Consultants are required to absent themselves from the room during the review of any application if their presence would constitute or appear to constitute a conflict of interest.
